# Naïve pooling of randomized and non-randomized evidence in network meta-analyses: a meta-epidemiological study protocol

**DOI:** 10.3389/fmed.2026.1868332

**Published:** 2026-06-15

**Authors:** Chongyang Zhao, Jia Song, Qin Wang, Li Li, Deying Kang

**Affiliations:** 1 Department of Evidence-Based Medicine and Clinical Epidemiology, West China Hospital, Sichuan University, Chengdu, China; 2 NHC Key Laboratory of Clinical Epidemiology and Evidence-Based Medicine, West China Hospital, Sichuan University, Chengdu, China; 3 School of Public Health and Preventive Medicine, Monash University, Melbourne, VIC, Australia; 4 Chengdu Fifth People's Hospital, Chengdu, China; 5 Department of Clinical Research Management, West China Hospital, Center of Biostatistics, Design, Measurement and Evaluation (CBDME), Sichuan University, Chengdu, China

**Keywords:** evidence synthesis, meta-epidemiology, methodology, network meta-analysis, non-randomized studies of interventions, randomized controlled trials

## Abstract

**Background:**

Network meta-analysis (NMA) can synthesize direct and indirect evidence across multiple interventions and is increasingly used to inform comparative-effectiveness decisions. When randomized controlled trials (RCTs) and non-randomized studies of interventions (NRSI) are jointly analyzed, naïve pooling of design-dissimilar evidence without design-aware modelling may violate exchangeability and distort treatment effect estimates and rankings.

**Methods and analysis:**

We will conduct a meta-epidemiological study of published clinical NMAs identified through systematic searches of Ovid MEDLINE(R) ALL, Embase via Ovid, EBM Reviews-Cochrane Database of Systematic Reviews via Ovid, and Web of Science Core Collection. Eligible reports will be full-text articles published between 1 January 2020 and 31 December 2025 that included both RCTs and at least one comparative NRSI in the review evidence base. The primary outcome will be the prevalence of naïve pooling in the main analysis. We will apply a prespecified four-category decision framework to distinguish unacknowledged naïve pooling, acknowledged but unmitigated naïve pooling, naïve main analysis with *post hoc* quantitative mitigation, and no naïve pooling in the main analysis. We will estimate prevalence with exact binomial 95% confidence intervals, assess whether naïve pooling has become more or less common over time, explore characteristics associated with naïve pooling or with not using quantitative methods to address design-related bias, summarize reporting completeness, protocol or registration availability, prespecification of design-handling methods, and availability of extractable paired alternative results, and evaluate empirical changes in effect direction, null-value crossing or statistical significance, and treatment ranking in articles reporting paired mixed-design and RCT-restricted or design-aware results.

**Discussion:**

This study will provide contemporary evidence on how published NMAs handle mixed randomized and non-randomized evidence, distinguish lack of risk recognition from failure to implement corrective analyses, and quantify how often naïve pooling may change conclusions. The protocol includes prespecified procedures to describe reporting completeness and to compare articles with and without extractable paired alternative analyses. The findings may inform methodological training, journal peer review, and evidence appraisal in guideline development.

**Systematic review registration:**

https://doi.org/10.17605/OSF.IO/AM2T6, Unique Identifier: 10.17605/OSF.IO/AM2T6.

## 1 Introduction

Network meta-analysis (NMA) allows simultaneous comparison of three or more competing interventions by combining direct and indirect evidence within a connected treatment network. Because NMA can estimate relative effects for comparisons that have not been evaluated head-to-head and can generate probabilistic treatment rankings, it has become a major evidence source for clinical guidelines, health technology assessment, and policy decisions (1–3).

Most NMAs have historically relied on randomized controlled trials (RCTs), which are less vulnerable to confounding in comparative-effectiveness research. However, there is increasing interest in incorporating non-randomized studies of interventions (NRSI) into NMAs, particularly when RCT evidence is sparse, when follow-up in RCTs is short, when rare or long-term harms are important, or when external validity is a central concern. At the same time, NRSI may carry selection bias, residual confounding, immortal-time bias, and other design-specific biases that are not eliminated by conventional random-effects models alone (4, 5).

Methodological work has shown that RCTs and NRSI should not automatically be treated as exchangeable pieces of evidence. Available alternatives to simple pooling include design-adjusted synthesis, use of NRSI as informative priors, three-level or design-specific hierarchical models, bias-adjusted models, and stratified or sensitivity analyses that explicitly evaluate whether conclusions depend on study design (5–7). NMA-specific confidence and risk-of-bias frameworks, including CINeMA, RoB NMA, and GRADE approaches for NMA, further emphasize the need to account for within-study bias, indirectness, intransitivity, and incoherence when mixed designs contribute to a network (8–10).

Despite these methodological developments, naïve pooling, defined here as joint synthesis of RCTs and NRSI in the same quantitative network model without any design-specific adjustment, stratification, weighting, or hierarchical treatment of study design, still appears in the published literature. A prior scoping review of earlier mixed-design NMAs found that 74% of eligible NMAs used naïve pooling, but that review covered a small historical sample and did not distinguish whether authors were unaware of the problem, acknowledged it without taking action, or attempted *post hoc* mitigation (11).

Adjacent evidence also supports the importance of this question. A recent meta-epidemiological study of pairwise meta-analyses found that integration of NRSI with RCTs was common and could materially change effect estimates or qualitative conclusions, but that study explicitly excluded NMAs (12). Simulation and methodological work has also shown that the choice among naïve synthesis, design-adjusted synthesis, informative priors, hierarchical models, and bias-corrected models can affect bias, precision, and coverage when non-randomized or real-world evidence is incorporated (5–7). These findings strengthen the rationale for studying contemporary NMAs specifically, where mixed-design evidence can influence not only direct comparisons but also indirect estimates, network coherence, and intervention rankings.

A contemporary meta-epidemiological assessment is therefore needed. Such an assessment should distinguish risk recognition from risk management, focus on the analysis that supports each article’s main conclusion, and measure impact by comparing mixed-design results with reported design-aware or RCT-restricted results within the same article whenever such paired results are available. The present protocol was developed to address these gaps.

The objectives of this study are fourfold: (i) to estimate the prevalence of naïve pooling in the main analysis of published clinical NMAs that included both RCTs and NRSI; (ii) to describe methodological handling patterns using a prespecified decision framework; (iii) to examine whether naïve pooling has become more or less common over time and to explore characteristics associated with naïve pooling or with not using quantitative methods to address design-related bias; and (iv) to quantify the empirical impact of naïve pooling by comparing mixed-design results with reported and extractable RCT-restricted or design-aware alternatives, where available.

## 2 Methods

### 2.1 Study design, reporting standards, and registration

This study is a systematic methodological review and meta-epidemiological survey of published clinical NMAs. The protocol was drafted in accordance with the PRISMA-P 2015 statement and elaboration (13, 14), and the completed study will be reported using PRISMA 2020 (15) and reporting guidance for meta-epidemiological methodology research (16). Items from the PRISMA extension for network meta-analysis and the Cochrane Handbook will be applied where relevant to NMA-specific methodological characteristics (2, 17).

This protocol was prospectively registered on the Open Science Framework on 28 April 2026 (doi: 10.17605/OSF.IO/AM2T6; https://osf.io/am2t6). The associated OSF project is available at: https://osf.io/2z73w. If PROSPERO accepts the project as within scope, the protocol will also be registered there. The registration identifier, timestamp, coding manual, and dated amendments will be reported in the final manuscript. Any deviations from this protocol will be transparently documented in the final report.

### 2.2 Eligibility criteria

Eligibility criteria are summarized in Table 1. The primary unit of inclusion will be the published article.

**Table 1:** Eligibility criteria for the meta-epidemiological review.

Domain	Include	Exclude
Report type	Peer-reviewed full-text systematic reviews with an NMA, mixed treatment comparison, or multiple-treatment meta-analysis of health interventions in humans.	Protocols, conference abstracts, letters, editorials, commentaries, narrative reviews, methodology papers, umbrella reviews, overviews of reviews, dissertations, and non-peer-reviewed reports.
Interventions and context	Any therapeutic or preventive health intervention compared in humans; all clinical specialties eligible.	Diagnostic test accuracy networks, prognostic-factor networks, etiology/exposure networks, prevalence networks, animal or *in vitro* studies, purely economic models without a clinical evidence synthesis.
Evidence composition	Review includes both RCTs and at least one comparative NRSI (for example cohort, case–control, quasi-experimental, non-randomized trial, comparative registry/database study) in the evidence base or quantitative synthesis.	Reviews including only RCTs, only NRSI, single-arm evidence only, or studies that mention NRSI in the search strategy but do not include any eligible NRSI in the final evidence base.
Publication period	First online or final publication between 1 January 2020 and 31 December 2025.	Articles first published before 2020 or after 2025.
Language	No language restriction at the search stage. Non-English reports will be translated where feasible using reviewer verification of machine-assisted translation or language expertise.	Reports for which adequate translation cannot be achieved after reasonable attempts will be listed as records awaiting classification and excluded from the primary quantitative summary.
Duplicates	The most complete report will be retained when the same review appears in multiple versions; supplementary appendices and protocols will be used to clarify methods.	Redundant secondary publications without additional methodological detail.

### 2.3 Information sources and search strategy

We will search Ovid MEDLINE(R) ALL, Embase via Ovid, EBM Reviews-Cochrane Database of Systematic Reviews via Ovid, and Web of Science Core Collection. The primary search will be conducted after registration and run in 2026 to maximize retrieval of articles first published in 2025 but indexed with delay. Eligibility will remain restricted to articles first published between 1 January 2020 and 31 December 2025.

Search strategies will combine terms for network meta-analysis (for example, “network meta-analysis,” “mixed treatment comparison,” and “multiple-treatment meta-analysis”) with a sensitive set of terms intended to capture observational, real-world, or non-randomized evidence. Because mixed-design NMAs are inconsistently indexed, the strategy will be iteratively refined against sentinel papers and supplemented by backward and forward citation tracking of all included studies and key methodological reviews. The full search strategies for all databases will be provided in the final manuscript; the complete Ovid MEDLINE search strategy is included in Appendix 1.

### 2.4 Study selection

All records will be imported into EndNote for de-duplication and then exported to a screening platform such as Rayyan or Covidence. Before formal screening, the review team will complete calibration exercises on 100 titles/abstracts and 20 full-text reports to refine the eligibility manual and harmonize interpretation of the inclusion criteria.

Two reviewers will independently screen titles and abstracts, then assess full texts of potentially eligible reports. Disagreements will be resolved by discussion; unresolved disagreements will be adjudicated by a third reviewer. Reasons for full-text exclusion will be recorded and reported in a PRISMA flow diagram.

### 2.5 Primary unit of analysis and selection of the main analysis

The primary unit of analysis will be the published article. Because some NMAs report several outcomes or more than one network, classification will focus on the analysis most clearly linked to the article’s principal conclusion. We will apply the following hierarchy: (i) the analysis explicitly labeled as primary or main by the authors; (ii) if no such label is provided, the first patient-important efficacy outcome emphasized in the abstract results or conclusion; and (iii) if the principal analysis remains unclear, the most prominently reported comparative-effectiveness network in the main text. For this purpose, “most prominently reported” will mean the network or outcome given greatest interpretive weight, judged in order by explicit language in the abstract or conclusion, placement as the first NMA result in the main text, presentation as a main-text network plot, league table, forest plot, or ranking table, and the amount of textual discussion devoted to the result.

Ambiguous multi-outcome articles will be adjudicated before mixed-design handling is coded. Two reviewers will independently apply the hierarchy and record the reason for selecting the main analysis. Disagreements will be resolved by discussion; unresolved cases will be referred to a third reviewer. If the main analysis remains unclear after adjudication, the article will be labeled unclear for main-analysis selection and handled in sensitivity analyses.

After the main analysis has been selected, the four-category framework in Table 2 will be used to characterize how that analysis handles mixed randomized and non-randomized designs. Separately, to assess the robustness of choosing a single main analysis, we will perform a worst-case article-level sensitivity analysis: if more than one main-text network meets the selection hierarchy and any such network uses naïve pooling, the article will be classified as having naïve pooling under this sensitivity rule. This rule will not be used to select the primary analysis; it will be used only to evaluate whether conclusions depend on the primary selection rule.

**Table 2:** Prespecified four-category framework for classifying mixed-design handling.

Category	Operational definition	Examples and important non-examples
A. Unacknowledged naïve pooling in the main analysis	The main analysis jointly synthesizes RCTs and NRSI in one model, with no explicit acknowledgment of design-related bias and no quantitative mitigation.	Example: one mixed-design network with standard heterogeneity assessment only. Non-example: mixed-design main analysis plus RCT-only sensitivity analysis.
B. Acknowledged but unmitigated naïve pooling in the main analysis	The main analysis is still naïve, but authors explicitly discuss confounding or design differences, apply separate RoB tools, or downgrade certainty, without any design-aware quantitative follow-up.	Example: authors use ROBINS-I and discuss residual confounding, but report only a single mixed-design network.
C. Naïve main analysis with post hoc quantitative mitigation	The main analysis is naïve, but at least one supplementary quantitative analysis tests robustness by study design.	Examples: RCT-only sensitivity analysis; separate RCT and NRSI networks; meta-regression by study design; design-adjusted, hierarchical, or bias-adjusted sensitivity analysis; exclusion or downweighting of NRSI at critical risk of bias.
D. No naïve pooling in the main analysis	The primary conclusions are based on a design-aware or design-restricted main analysis from the outset.	Examples: RCT-only primary NMA; design-adjusted synthesis; hierarchical model with study design; NRSI used only as informative priors. Node-splitting alone is not sufficient for this category.

### 2.6 Data extraction and coding manual

Data extraction will be performed independently and in duplicate using a piloted electronic form. The coding manual will be developed *a priori* and revised only during calibration; any changes after pilot testing will be dated and archived. Reviewers will extract data from the main text, supplementary files, appendices, publicly accessible protocols, and registration records when available.

If an essential methodological detail remains unclear after reviewing all available materials, we will contact the corresponding author once with a focused query limited to targeted factual clarification, rather than a request for new analyses, reanalysis, or substantive reinterpretation. A prespecified 14-calendar-day response window will be used, consistent with published meta-epidemiological author-contact practice using an approximately two-week response period (18). This interval is intended to give authors a reasonable opportunity to verify limited methodological details while preventing indefinite delays and maintaining a reproducible, equally applied coding process. If no response is received within 14 calendar days, the variable will be coded as “not reported” or “unclear” according to prespecified rules. Non-response will be recorded only as a reporting-status variable and will not be interpreted as evidence of publication bias, selective reporting, or any selection mechanism. Unresolved missing information may affect classification of author acknowledgment, quantitative mitigation, main-analysis selection, and eligibility for the empirical-impact subset; these variables will therefore be flagged, summarized, and examined in sensitivity analyses rather than inferred. Table 3 lists the minimum variables to be extracted.

**Table 3:** Core data items and operational definitions.

Domain	Variables	Operational notes
Bibliographic data	Title, journal, DOI, year of first publication, clinical specialty, journal quartile/metric at extraction time if available.	Publication year will be based on first online publication when reported.
Review team and reporting features	Registration status, funding source, corresponding author region, presence of a methods-affiliated author.	A methods-affiliated author will be defined a priori by affiliation terms such as biostatistics, epidemiology, evidence synthesis, or health research methods.
Network characteristics	Number of interventions, number of included studies, number and proportion of RCTs and NRSI, outcomes analyzed, software, Bayesian versus frequentist framework, ranking metric.	The proportion of NRSI will be calculated as NRSI / total included comparative studies where possible.
Use of NRSI	Whether NRSI contributed to the main network, only to sensitivity analyses, only to selected outcomes, or were used to connect an otherwise disconnected network.	Comparative NRSI must contribute at least to the evidence base to meet eligibility; single-arm evidence will not qualify.
Risk-of-bias and certainty methods	Tools used for RCTs and NRSI (for example RoB 2 and ROBINS-I), whether certainty was assessed (for example CINeMA or GRADE).	These variables will be summarized descriptively and will not be included as covariates in association models if they overlap conceptually with the outcome definition.
Design handling strategy	Main and supplementary analytic approaches for mixed designs, including naïve pooling, RCT-only primary analysis, study-design subgroup analysis, meta-regression by design, design-adjusted synthesis, priors from NRSI, hierarchical models, or bias-adjusted models.	Operational definitions are provided in Section 2.7 and Table 2.
Empirical impact subset	Availability of paired mixed-design and RCT-restricted or design-aware results for the same comparison/outcome; authors’ stated alternative-analysis approach, with examples including RCT-only analysis, study-design subgroup analysis, meta-regression by design, design-adjusted or bias-adjusted NMA, hierarchical design-specific model, use of NRSI as informative priors, or design-based exclusion/downweighting; change in effect direction; change in null crossing/statistical significance; change in top-ranked intervention; and whether the article is included in the empirical-impact subset.	Top-ranked intervention will be based on SUCRA, p-score, posterior ranking probabilities, or the intervention explicitly described as best if no metric is reported. Articles with and without paired results will be compared descriptively to contextualize the empirical-impact subset.
Reporting-completeness, availability, and extractability descriptors	Protocol or registration availability; whether design-handling methods were prespecified; consistency between available protocol and published report; completeness of reporting of design-aware analyses; need for supplementary materials to classify design handling; journal quartile/metric, funding source, and methods-affiliated authorship.	These descriptors will be used to characterize reporting completeness, protocol or registration availability, prespecification of design-handling methods, consistency between available protocols and published reports, and the availability and extractability of design-aware or paired alternative results. They will provide contextual information for interpreting classification and the empirical-impact subset, and will not be used to assess publication bias, detect selective reporting, or infer any publication-, journal-, or author-level selection mechanism.

### 2.7 Operational definitions and decision framework

Naïve pooling will be defined as joint quantitative synthesis of RCT and NRSI effect estimates within the same network model or pairwise model without any explicit treatment of study design as a source of non-exchangeability. In other words, a mixed-design random-effects NMA remains naïve if the model neither stratifies by design, nor includes a study-design covariate, nor downweights or adjusts NRSI for expected bias, nor treats NRSI as prior information, nor uses a hierarchical design-specific structure.

Several practices will not be considered adequate quantitative mitigation by themselves. These include a generic random-effects model, standard global or local inconsistency checks, node-splitting conducted without any study-design dimension, separate narrative discussion of bias, or certainty downgrading alone. These practices may indicate awareness of risk, but they do not by themselves address the non-randomized component of the quantitative synthesis.

By contrast, design-aware handling strategies will include any one of the following: RCT-only primary analysis with NRSI reserved for supplementary use; separate analyses by study design; subgroup analysis or meta-regression using study design as a covariate; design-adjusted or bias-adjusted NMA; hierarchical models with study-design levels; use of NRSI as informative priors; or any prespecified exclusion/downweighting rule that explicitly depends on study design or NRSI bias status.

### 2.8 Reporting-completeness, availability, and extractability considerations

Because the unit of analysis is the published NMA article rather than patient-level intervention effects, conventional funnel-plot assessment of small-study effects or publication bias will not be applicable to the primary methodological outcome. We will therefore record observable indicators of reporting completeness, availability, and extractability relevant to classification of mixed-design handling and interpretation of the empirical-impact subset. These indicators will include whether a protocol or registration was available, whether handling of RCT and NRSI evidence was prespecified, whether published methods were consistent with the protocol when available, whether analyses that addressed design-related bias were reported with extractable results or only mentioned narratively, and whether supplementary materials or author clarification were needed for classification.

We will summarize these indicators descriptively. They will be used as contextual information about reporting transparency, protocol or registration availability, prespecification, and extractability of paired alternative results. They will not be used to assess publication bias, detect selective reporting, or infer any publication-, journal-, or author-level selection mechanism.

### 2.9 Outcomes

Primary outcome: the prevalence of naïve pooling in the main analysis, defined as the proportion of eligible articles classified as category A, B, or C among all included publications.

Key secondary outcomes: (i) the prevalence of unadjusted naïve pooling without any quantitative mitigation (categories A + B); (ii) the distribution of the four categories overall and by publication year; (iii) the frequency of specific design-aware handling strategies; (iv) associations between *a priori* selected characteristics and naïve pooling or not using quantitative methods to address design-related bias; and (v) the empirical impact of naïve pooling in the subset of articles that report both mixed-design and design-aware or RCT-restricted results for the same comparison or outcome.

For the empirical-impact subset, we will extract paired mixed-design and alternative results reported for the same comparison, outcome, and time point. For each alternative analysis, we will record the authors’ stated approach to handling NRSI, the effect estimate and interval when reported, the top-ranked intervention when available, and whether the alternative result changes effect direction, null-value crossing, statistical significance as reported by the authors, or the top-ranked intervention.

To describe the composition of the empirical-impact subset and examine whether it differs from other eligible mixed-design NMAs, we will compare articles with eligible paired empirical-impact results with eligible articles without such results on publication year, clinical specialty, journal metric category, total number of included studies, proportion of NRSI, and the four-category design-handling classification. We will also summarize the reasons paired results are unavailable, such as absence of any design-aware analysis, incomplete reporting of alternative results, or non-comparable outcomes.

### 2.10 Statistical analysis

No formal sample size calculation is required because the aim is to identify the full census of eligible articles within the prespecified time window. All analyses will be conducted in R (R Foundation for Statistical Computing, Vienna, Austria). The nominal significance threshold will be two-sided alpha = 0.05, with emphasis placed on effect estimates and confidence intervals rather than *p*-values alone.

We will summarize categorical variables using frequencies and percentages. Continuous variables will be summarized using means, standard deviations, medians, and interquartile ranges. Overall prevalence estimates will be presented with exact binomial 95% confidence intervals (19). Year-specific proportions will be displayed graphically using stacked bar charts and line plots with confidence intervals.

Temporal trends in the primary binary outcome (naïve pooling in the main analysis: yes versus no) will be examined using logistic regression with publication year entered as a continuous variable. We will report the odds ratio for naïve pooling per 1-year increase in publication year. As a sensitivity analysis, we will assess non-linearity using restricted cubic splines if the number of eligible studies is sufficient. Trends across the four-category framework will be examined using multinomial logistic regression with category D as the reference.

To explore *a priori* selected characteristics associated with mixed-design handling, two exploratory association models will be specified: one comparing categories A + B versus categories C + D, and one comparing categories A + B + C versus category D. Candidate characteristics will be selected a priori for conceptual relevance and expected extractability, and will cover publication timing, clinical area, journal and author features, protocol/registration status, analytic framework, evidence-base size, NRSI proportion and network role, and funding source. Final association models will be restricted to characteristics with sufficient completeness and cell counts; sparse or poorly reported characteristics will be summarized descriptively, and variables that form part of the outcome definition will not be included as covariates.

If sparse data or separation occur, we will use Firth penalized logistic regression (20, 21). The number of covariates included in association models will be restricted according to outcome frequency and effective sample size; if events per parameter are limited, we will use reduced prespecified models and avoid overfitting (22). Missingness in candidate characteristics will be summarized. Complete-case analysis will be primary; if missingness for any key characteristic exceeds 10% and the data structure is compatible with missing-at-random assumptions, multiple imputation by chained equations will be explored in sensitivity analyses (23, 24).

Empirical-impact results will be summarized descriptively at the article level and, where multiple paired alternatives are reported, according to the authors’ reported alternative-analysis approach. Counts and proportions will be reported for changes in effect direction, null-value crossing or statistical significance, and top-ranked intervention. Numerical changes in effect estimates or interval widths will be summarized only when the paired estimates are sufficiently extractable and comparable.

### 2.11 Inter-rater reliability, ambiguity management, and sensitivity analyses

Cohen’s kappa with 95% confidence intervals will be calculated for title/abstract screening, full-text eligibility assessment, main-analysis selection, and the four-category classification of mixed-design handling (25). Any persistent ambiguities in classification will be adjudicated by a senior reviewer using the coding manual. If ambiguity remains after adjudication, the article will be labeled “unclear” for that item and excluded from the relevant primary analysis; sensitivity analyses will then reclassify unclear cases under best-case and worst-case assumptions.

Prespecified sensitivity analyses will include: (i) restricting the sample to articles with a clearly labeled primary outcome or main analysis; (ii) applying the worst-case article-level coding rule described in Section 2.5; (iii) excluding articles in which NRSI were identified but only narratively discussed; (iv) excluding articles with unresolved methodological ambiguity; and (v) reclassifying unclear cases under best-case and worst-case assumptions.

### 2.12 Ethics and dissemination

Because this study will use data extracted from published literature and will not involve individual patient data or direct participant contact, formal ethics approval is not required. Nevertheless, the review team will follow principles of transparent, reproducible research.

The extraction form, coding manual, analytic code, and article-level dataset generated for this review will be made publicly available on OSF or a comparable repository at the time of publication, subject to copyright limitations for full-text articles. Findings will be submitted to a peer-reviewed journal and presented at relevant evidence-synthesis or clinical methodology conferences.

## 3 Discussion

This protocol addresses a methodological question of direct relevance to the credibility of contemporary evidence synthesis. In many clinical areas, NRSI are increasingly invoked to expand networks, connect sparse comparisons, or provide real-world context. However, the act of combining RCTs and NRSI is not methodologically neutral. If mixed designs are synthesized as though they were fully exchangeable, the resulting network may inherit confounding and design-specific biases while presenting them with the appearance of precision and comprehensiveness.

The main strengths of this protocol are its focus on the analysis that supports each article’s published conclusion, its distinction between risk recognition and risk management, its prespecified decision framework for classifying mixed-design handling, and its plan to evaluate empirical within-article consequences of naïve pooling when paired alternative analyses are reported. Using within-article contrasts, rather than comparisons across unrelated reviews, may reduce differences in topic, population, intervention set, and outcome definition when estimating empirical impact.

Several limitations should be acknowledged in advance. First, this review relies on what authors report in published articles and supplements; unreported analytic decisions cannot be recovered with certainty. Second, the empirical-impact analysis will necessarily be limited to articles that report extractable paired mixed-design and RCT-restricted or design-aware results; these articles may be more methodologically transparent than other eligible NMAs. We will describe differences between articles with and without paired results, but residual selection bias may remain. Third, classification of author intent, main analysis, and design handling may remain difficult in multi-outcome or incompletely reported articles. Fourth, reporting-completeness, availability, and extractability indicators are based on published records and author clarifications where available; they can describe reporting transparency and the availability of extractable methodological information but cannot establish whether unreported analyses or methodological decisions were selectively withheld, and cannot be used to infer publication-, journal-, or author-level selection mechanisms. The planned adjudication process, sensitivity analyses, and comparisons of articles with and without paired results are intended to quantify the influence of these limitations on classification and interpretation.

Notwithstanding these limitations, the study has the potential to produce actionable evidence. If naïve pooling remains common, or if acknowledgment without action predominates, the findings could support clearer journal standards, stronger peer-review expectations, and more targeted training for review authors. Conversely, if design-aware handling has become more common, the study will document that methodological uptake and help identify which practices are becoming normative.

## 4 Conclusion

This meta-epidemiological study will provide an updated, operationally rigorous assessment of how published NMAs handle mixed randomized and non-randomized evidence. By estimating prevalence, distinguishing methodological handling patterns, exploring characteristics associated with naïve pooling or lack of quantitative methods to address design-related bias, and quantifying empirical impact where paired mixed-design and alternative results are reported, the review is designed to generate evidence that is directly relevant to methodological guidance, journal editorial standards, and evidence-based decision-making. Reporting-completeness, availability, and extractability descriptors will provide descriptive context for interpreting these findings.

## Appendix

Appendix 1: Ovid MEDLINE search strategy.

Search platform: Ovid. Database: Ovid MEDLINE(R) ALL.

1. Network Meta-Analysis.pt. or network meta-analysis as topic/.
2. ((network adj3 (meta-analy* or metaanaly*)) or “mixed treatment comparison*” or “multiple treatment comparison*” or “multiple-treatment meta-analysis” or “multiple-treatment meta-analyses” or “indirect treatment comparison*” or “indirect comparison*” or SUCRA or rankogram* or “surface under the cumulative ranking”).ti,ab,kf
3. 1 or 2
4. Observational Study.pt. or exp. Observational Studies as Topic/ or exp. Cohort Studies/ or exp. Case–Control Studies/ or exp. Registries/ or Non-Randomized Controlled Trials as Topic/
5. (observational or cohort* or case–control* or “case control*” or registry or registries or real-world or “real world” or “real-world evidence” or “real-world data” or nonrandomized or nonrandomised or “non-randomized” or “non-randomised” or “non randomized” or “non randomised” or NRSI or “non-randomized studies of interventions” or ROBINS-I or “ROBINS I” or claims or “administrative data” or “electronic health record*” or (database* adj3 (study or studies or cohort* or registry or registries or claims or administrative or “electronic health record*”)) or propensity or “Newcastle-Ottawa” or quasi-experiment* or retrospective or prospective).ti,ab,kf
6. 4 or 5
7. 3 and 6
8. limit 7 to yr. = “2020–2025”

## References

[1] Caldwell DM Ades AE Higgins JPT. Simultaneous comparison of multiple treatments: combining direct and indirect evidence. BMJ. (2005) 331:897–900. doi: 10.1136/bmj.331.7521.897, 16223826 PMC1255806

[2] Hutton B Salanti G Caldwell DM Chaimani A Schmid CH Cameron C . The PRISMA extension statement for reporting of systematic reviews incorporating network meta-analyses of health care interventions: checklist and explanations. Ann Intern Med. (2015) 162:777–84. doi: 10.7326/M14-2385, 26030634

[3] Ades AE Welton NJ Dias S Phillippo DM Caldwell DM. Twenty years of network meta-analysis: continuing controversies and recent developments. Res Synth Methods. (2024) 15:702–27. doi: 10.1002/jrsm.1700, 38234221

[4] Cameron C Fireman B Hutton B Clifford T Coyle D Wells G . Network meta-analysis incorporating randomized controlled trials and non-randomized comparative cohort studies for assessing the safety and effectiveness of medical treatments: challenges and opportunities. Syst Rev. (2015) 4:147. doi: 10.1186/s13643-015-0133-0, 26537988 PMC4634799

[5] Efthimiou O Mavridis D Debray TPA Samara M Belger M Siontis GCM . Combining randomized and non-randomized evidence in network meta-analysis. Stat Med. (2017) 36:1210–26. doi: 10.1002/sim.7223, 28083901

[6] Hussein H Abrams KR Gray LJ Anwer S Dias S Bujkiewicz S. Hierarchical network meta-analysis models for synthesis of evidence from randomised and non-randomised studies. BMC Med Res Methodol. (2023) 23:97. doi: 10.1186/s12874-023-01925-5, 37087450 PMC10122363

[7] Yao M Wang Y Ren Y Jia Y Zou K Li L . Comparison of statistical methods for integrating real-world evidence in a rare events meta-analysis of randomized controlled trials. Res Synth Methods. (2023) 14:689–706. doi: 10.1002/jrsm.1648, 37309821

[8] Nikolakopoulou A Higgins JPT Papakonstantinou T Chaimani A Del Giovane C Egger M . CINeMA: an approach for assessing confidence in the results of a network meta-analysis. PLoS Med. (2020) 17:e1003082. doi: 10.1371/journal.pmed.1003082, 32243458 PMC7122720

[9] Lunny C Higgins JPT White IR Dias S Hutton B Wright JM . Risk of Bias in network Meta-analysis (RoB NMA) tool. BMJ. (2025) 388:e079839. doi: 10.1136/bmj-2024-079839, 40101916 PMC11915405

[10] Puhan MA Schünemann HJ Murad MH Li T Brignardello-Petersen R Singh JA . A GRADE working group approach for rating the quality of treatment effect estimates from network meta-analysis. BMJ. (2014) 349:g5630. doi: 10.1136/bmj.g5630, 25252733

[11] Zhang K Arora P Sati N Béliveau A Troke N Veroniki AA . Characteristics and methods of incorporating randomized and nonrandomized evidence in network meta-analyses: a scoping review. J Clin Epidemiol. (2019) 113:1–10. doi: 10.1016/j.jclinepi.2019.04.023, 31059803

[12] Mei F Yao M Wang Y Huan J Ma Y Li G . Integration of non-randomized studies with randomized controlled trials in meta-analyses of clinical studies: a meta-epidemiological study on effect estimation of interventions. BMC Med. (2024) 22:571. doi: 10.1186/s12916-024-03778-1, 39623370 PMC11613474

[13] Moher D Shamseer L Clarke M Ghersi D Liberati A Petticrew M . Preferred reporting items for systematic review and meta-analysis protocols (PRISMA-P) 2015 statement. Syst Rev. (2015) 4:1. doi: 10.1186/2046-4053-4-1, 25554246 PMC4320440

[14] Shamseer L Moher D Clarke M Ghersi D Liberati A Petticrew M . Preferred reporting items for systematic review and meta-analysis protocols (PRISMA-P) 2015: elaboration and explanation. BMJ. (2015) 349:g7647. doi: 10.1136/bmj.g7647, 25555855

[15] Page MJ McKenzie JE Bossuyt PM Boutron I Hoffmann TC Mulrow CD . The PRISMA 2020 statement: an updated guideline for reporting systematic reviews. BMJ. (2021) 372:n71. doi: 10.1136/bmj.n71, 33782057 PMC8005924

[16] Murad MH Wang Z. Guidelines for reporting meta-epidemiological methodology research. Evid Based Med. (2017) 22:139–42. doi: 10.1136/ebmed-2017-110713, 28701372 PMC5537553

[17] Higgins J. P. T. Thomas J. Chandler J. Cumpston M. Li T. Page M. J. . Cochrane Handbook for Systematic Reviews of Interventions Version 6.5 (Updated August 2024). Cochrane; (2024). Available online at: https://training.cochrane.org/handbook (Accessed April 28, 2026).

[18] Saquib N Saquib J Ioannidis JPA. Practices and impact of primary outcome adjustment in randomized controlled trials: meta-epidemiologic study. BMJ. (2013) 347:f4313. doi: 10.1136/bmj.f4313, 23851720 PMC3709831

[19] Clopper CJ Pearson ES. The use of confidence or fiducial limits illustrated in the case of the binomial. Biometrika. (1934) 26:404–13. doi: 10.1093/biomet/26.4.404

[20] Firth D. Bias reduction of maximum likelihood estimates. Biometrika. (1993) 80:27–38. doi: 10.1093/biomet/80.1.27

[21] Heinze G Schemper M. A solution to the problem of separation in logistic regression. Stat Med. (2002) 21:2409–19. doi: 10.1002/sim.1047, 12210625

[22] Vittinghoff E McCulloch CE. Relaxing the rule of ten events per variable in logistic and cox regression. Am J Epidemiol. (2007) 165:710–8. doi: 10.1093/aje/kwk052, 17182981

[23] van Buuren S Groothuis-Oudshoorn K. Mice: multivariate imputation by chained equations in R. J Stat Softw. (2011) 45:1–67. doi: 10.18637/jss.v045.i03

[24] White IR Royston P Wood AM. Multiple imputation using chained equations: issues and guidance for practice. Stat Med. (2011) 30:377–99. doi: 10.1002/sim.4067, 21225900

[25] Cohen J. A coefficient of agreement for nominal scales. Educ Psychol Meas. (1960) 20:37–46. doi: 10.1177/001316446002000104

